# Aseptische Osteonekrose des medialen Femurkondylus bei einem Patienten mit akuter SARS-CoV-2 Infektion

**DOI:** 10.1007/s00113-021-01082-8

**Published:** 2021-09-29

**Authors:** Andreas Thannheimer, Christian von Rüden

**Affiliations:** 1 Abteilung Unfallchirurgie, Sportorthopädie und Kindertraumatologie, Klinikum Garmisch-Partenkirchen, Garmisch-Partenkirchen, Deutschland; 2 Abteilung Rekonstruktive Unfallchirurgie und Orthopädie, BG Unfallklinik Murnau, Professor-Küntscher-Str. 8, 82418 Murnau, Deutschland; 3 Institut für Biomechanik, BG Unfallklinik Murnau, Murnau, Deutschland; 4 Universitätsinstitut für Biomechanik, Paracelsus Medizinische Privatuniversität, Salzburg, Österreich

## Anamnese und Befund

Ein 72-jähriger Mann stellte sich während der „Coronavirus-disease-2019“(COVID-19)-Pandemie im September 2020 erstmalig mit den klassischen klinischen Symptomen einer Innenmeniskusläsion des linken Kniegelenks im Klinikum Garmisch-Partenkirchen vor. Eigenanamnestisch waren die Beschwerden wenige Wochen zuvor erstmals infolge einer Bagatellverletzung ohne adäquates Trauma aufgetreten.

## Diagnose

**Figure 1:**
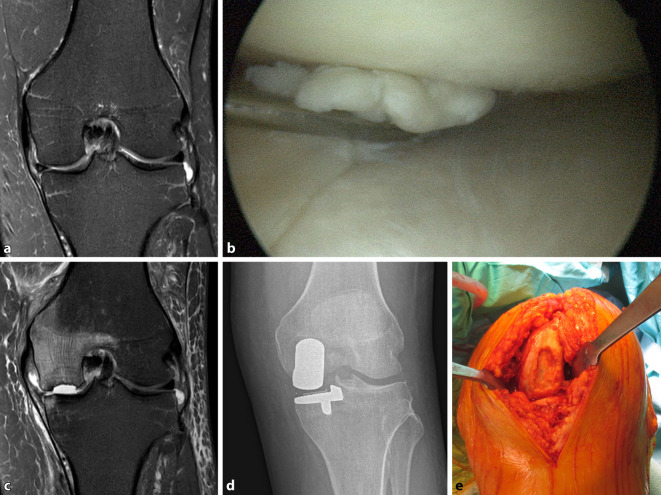

Eine im Vorfeld angefertigte Magnetresonanztomographie (MRT) bestätigte den klinischen Befund in Form einer Konturunterbrechung im Hinterhorn des Innenmeniskus (Abb. 1a). Weitere relevante pathologische Befunde fanden sich nicht.

## Therapie

Im Oktober 2020 wurde ambulant eine komplikationslose arthroskopische Teilresektion des Innenmeniskushinterhorns durchgeführt (Abb. 1b).

## Verlauf

Sechzehn Tage nach dem Eingriff wurde der Patient positiv auf das „severe acute respiratory syndrome coronavirus 2“ (SARS-CoV-2) getestet. Der Überträger wurde im familiären Umfeld vermutet. Eine Kortikoidtherapie war bei milder klinischer Symptomatik nicht erforderlich. Aufgrund in der aktuellen Literatur beschriebener möglicher vaskulärer Begleitveränderungen [1, 2] nahm der Patient prophylaktisch einmal täglich 100 mg Acetylsalicylsäure ein. Nach unkompliziertem Verlauf der SARS-CoV-2-Infektion stellte er sich wegen anhaltender Beschwerden am operierten Kniegelenk Ende November 2020 erneut bei seinem Operateur vor. Klinisch zeigte sich nun eine deutliche Ergussbildung, begleitet von einer Minderbelastbarkeit. Die Belastungsschmerzen waren eindeutig auf den anteromedialen Gelenkabschnitt projizierbar. Unter der Vorstellung eines noch bestehenden postoperativen Reizzustandes wurde zunächst ein abwartendes Verhalten mit lastfreier Übungsbehandlung vereinbart. Bei einige Tage später akut zunehmenden klinischen Beschwerden mit Blockadephänomen wurde Mitte Dezember 2020 eine MRT-Kontrolluntersuchung durchgeführt. Diese zeigte eine fortgeschrittene Osteonekrose des medialen Femurkondylus mit Ablösung eines großen osteochondralen Fragments in der Hauptbelastungszone (Abb. 1c). Bei anhaltend aufgehobener Belastbarkeit wurde die dringliche Indikation zum unilateralen Gelenkoberflächenersatz noch Mitte Dezember 2020 gestellt (Abb. 1d). Der intraoperative klinische Befund (Abb. 1e) bestätigte den zuvor erhobenen MRT-Befund. Das histologische Bild eines während der Prothesenimplantation gewonnenen Knochenstanzbiopsats bestätigte das Vorliegen einer aseptischen Osteonekrose.

## Fallanalyse

Die Genese einer Osteonekrose ist nur selten sicher zu klären. Es lassen sich die avaskuläre, die aseptische und die subchondrale avaskuläre Nekrose voneinander unterscheiden [3]. Zur raschen Ausbildung der hier vorliegenden aseptischen Osteonekrose am medialen Femurkondylus kamen vier potenzielle Ursachen in Betracht:

1. Idiopathische avaskuläre Osteonekrose des älteren Menschen (M. Ahlbäck) [3] bei zufällig gleichzeitigem Ablauf einer akuten SARS-CoV-2-Infektion. Außer männlichem Geschlecht und fortgeschrittenem Alter wies der Patient allerdings keine weiteren Risikofaktoren für eine idiopathische Entstehung der Osteonekrose auf.
2. Komplikation der Kniegelenkarthroskopie. Das gelegentliche Auftreten eines primär radiologisch nicht sichtbaren Knochenmarködems nach Kniegelenkarthroskopie ist bekannt und wurde erstmals von Brahme et al. 1991 beschrieben [4, 5]. Das Ausmaß der Nekrose macht dies jedoch im aktuellen Fall eher unwahrscheinlich.
3. Nebenwirkung der Kortikoidtherapie einer akuten SARS-CoV-2-Infektion. Schon während der von SARS-CoV‑1 verursachten SARS-Pandemie der Jahre 2002–2004 wurde das vermehrte Auftreten von Osteonekrosen insbesondere des Hüftkopfes beschrieben [6]. Die Osteonekrosen wurden auf die üblicherweise angewendete Kortikoidtherapie zur Behandlung der akuten SARS-Infektion zurückgeführt und als deren Nebenwirkung bewertet [7, 8]. Der Effekt wurde dosisabhängig, aber auch abhängig von der Therapiedauer und bei der Verwendung von mehr als einem Steroid beobachtet [9]. Unter Bezug auf diese Beobachtungen wird aktuell vor einem unkritischen Einsatz von Kortikosteroiden bei der Akuttherapie der SARS-CoV-2-Infektion gewarnt [10, 11]. Aufgrund des milden klinischen Verlaufs seiner SARS-CoV-2-Infektion hatte der vorgestellte Patient keine Kortikoidtherapie erhalten. Eine Nebenwirkung dieser Therapie als mögliche Ursache der Osteonekrose kam somit hier nicht infrage.
4. Vaskuläre Komplikation der akuten SARS-CoV-2-Infektion. Die Kombination aus Hyperkoagulabilität, Leukozytenaggregation und Gefäßinflammation bei einer SARS-CoV-2-Infektion kann den mikrovaskulären Blutfluss im Knochen beeinträchtigen und zur Entwicklung einer Osteonekrose beitragen [2]. Entsprechende vaskuläre Komplikationen wurden bereits beschrieben [1, 8], allerdings bisher nicht am medialen Femurkondylus.

## Fazit für die Praxis

- Neben einer grundsätzlich möglichen idiopathischen Genese ließ der beobachtete Krankheitsverlauf bei fehlender Kortikoidtherapie der akuten SARS-CoV-2-Infektion auch an eine vaskuläre Komplikation im Rahmen der Infektion als potenzielle Ursache der aseptischen Osteonekrose des medialen Femurkondylus denken.
- Anhand dieser Fallbeschreibung soll zur Diskussion angeregt und in Erfahrung gebracht werden, ob auch andernorts schon eine akute Osteonekrose des medialen Femurkondylus in direktem zeitlichem Zusammenhang mit einer akuten SARS-CoV-2-Infektion beobachtet wurde.
